# Analysis of *Mycobacterium ulcerans*-specific T-cell cytokines for diagnosis of Buruli ulcer disease and as potential indicator for disease progression

**DOI:** 10.1371/journal.pntd.0005415

**Published:** 2017-02-27

**Authors:** Norman Nausch, Daniel Antwi-Berko, Yusif Mubarik, Kabiru Mohammed Abass, Wellington Owusu, Ellis Owusu-Dabo, Linda Batsa Debrah, Alexander Yaw Debrah, Marc Jacobsen, Richard O. Phillips

**Affiliations:** 1 Pediatric Pneumology and Infectious Diseases Group, Department of General Pediatrics, Neonatology and Pediatric Cardiology, University Children’s Hospital, Heinrich-Heine University, Dusseldorf, Germany; 2 Kumasi Centre for Collaborative Research in Tropical Medicine, KNUST, Kumasi, Ghana; 3 Agogo Presbyterian Hospital, Agogo, Ghana; 4 Department of Global Health, School of public health, College of Health Sciences, KNUST, Kumasi, Ghana; 5 School of Medical Sciences, Kwame Nkrumah University of Science and Technology, Kumasi, Ghana; 6 Faculty of Allied Health Sciences of Kwame Nkrumah University of Science and Technology, Kumasi, Ghana; University of Tennessee, UNITED STATES

## Abstract

**Background:**

Buruli ulcer disease (BUD), caused by *Mycobacterium (M*.*) ulcerans*, is the third most common mycobacterial disease after tuberculosis and leprosy. BUD causes necrotic skin lesions and is a significant problem for health care in the affected countries. As for other mycobacterial infections, T cell mediated immune responses are important for protection and recovery during treatment, but detailed studies investigating these immune responses in BUD patients are scarce. In this study, we aimed to characterise *M*. *ulcerans*-specific CD4+ T cell responses in BUD patients and to analyse specific cytokine-producing T cells in the context of disease severity and progression.

**Methodology/Principal findings:**

For this case-control study, whole blood samples of BUD patients (N = 36, 1.5–17 years of age) and healthy contacts (N = 22, 3–15 years of age) were stimulated with antigen prepared from *M*. *ulcerans* and CD4+ T cells were analysed for the expression of TNFα, IFNγ and CD40L by flow cytometry. The proportions and profile of cytokine producing CD4+ T cells was compared between the two study groups and correlated with disease progression and severity. Proportions of cytokine double-positive IFNγ+TNFα+, TNFα+CD40L+, IFNγ+CD40L+ (p = 0.014, p = 0.010, p = 0.002, respectively) and triple positive IFNγ+TNFα+CD40L+ (p = 0.010) producing CD4+ T cell subsets were increased in BUD patients. In addition, TNFα+CD40L-IFNγ- CD4+ T cells differed between patients and controls (p = 0.034). TNFα+CD40L-IFNγ- CD4+ T cells were correlated with lesion size (p = 0.010) and proportion were higher in ‘slow’ healers compared to ‘fast healers’ (p = 0.030).

**Conclusions:**

We were able to identify *M*. *ulcerans*-specific CD4+ T cell subsets with specific cytokine profiles. In particular a CD4+ T cell subset, producing TNFα but not IFNγ and CD40L, showed association with lesion size and healing progress. Further studies are required to investigate, if the identified CD4+ T cell subset has the potential to be used as biomarker for diagnosis, severity and/or progression of disease.

## Author summary

Buruli ulcer disease (BUD) is a devastating skin disease characterised by nodules, plaque or oedema at early stages, which progress to a characteristic form of ulcer. Without treatment the disease can cause broader tissue destruction, affect bones and cause permanent disabilities. BUD is treated with a combination of two antibiotics over a period of eight weeks. Overall this treatment scheme is effective, but duration of the healing process varies strongly. Specific immune responses are of major importance for controlling other mycobacterial infections (e.g. tuberculosis), but are also used for immune-based diagnostic tests. However, detailed knowledge of specific immune responses in BUD patients is missing. The aim of this study was to characterise the specific cytokine response of CD4+ T helper cells in BUD patients and to correlate specific T cell subsets to clinical disease progression. Using flow cytometry on *Mycobacterium ulcerans*-stimulated blood samples, we identified specific cytokine-secreting T cell subsets, induced in BUD patients. Proportions of a CD4+ T cell subset, producing a single cytokine were positively associated with lesion size and healing time. Results of this study provide deeper insights in the specific immune response in BUD patients and identify a potential indicator for early diagnosis and disease progression.

## Introduction

Buruli ulcer disease (BUD), caused by *Mycobacterium ulcerans*, is a neglected tropical disease with reported cases in 33 subtropical and tropical countries [1]. The majority of cases have been recorded in 12 countries, mainly in Western and Central Africa with Ghana among those countries where BUD is a significant problem for public health [1]. Notably about half of all cases are diagnosed in children and adolescents with dramatic consequences for their health and social life.

At early stages BUD is characterised by painless, subcutaneous papules, nodules, plaques or oedemas. Without treatment, most lesions enlarge progressively and ulcerate developing undermined skin borders [2, 3]. The disease can eventually destroy tissues, affect bones leading to deformation and may cause permanent disabilities [4, 5]. However, in some cases non-ulcerative lesions are stable and do not progressively enlarge or ulcerate.

Treatment outcome has substantially improved with the introduction of a combination therapy with rifampicin (given orally) and streptomycin (intramuscular injection) [1, 6, 7]. However an effective vaccine against infection is currently not available. Studies on protection of Bacillus Calmette-Guérin (BCG) vaccination led to contradictory results [8–10].

In the absence of protective vaccines and with limited knowledge about the exact mode of transmission [11], prevention of BUD is currently not feasible [12]. Therefore disease management relies on early case detection, reliable diagnosis and on early effective treatment. Classical methods of diagnosis such as isolation by *M*. *ulcerans* culture or smear microscopy of acid-fast bacilli take many weeks and/or lack sensitivity [13, 14]. The current gold standard for diagnosis is based on PCR dependent detection of the *M*. *ulcerans*-specific insertion sequence *IS2404* from lesion specimens. This test shows high sensitivity and specificity, but is not helpful in monitoring disease progression.

Immune-based assays provide a reliable tool for the detection of other mycobacterial diseases. In tuberculosis, Interferon-gamma release assays (IGRAs) are important for the diagnosis of *Mycobacterium tuberculosis* infections [15]. IGRAs detect interferon-gamma (IFNγ) following *in vitro* stimulation with specific antigens. IFNγ is either quantified from plasma of cultured blood (QuantiFERON) or by detecting IFNγ producing T cells (T-SPOT.*TB*). A comparable test based on the activation of *M*. *ulcerans*-specific T cells is currently not available. However, it is tempting to speculate, that such an immune based test could be beneficial for early diagnosis, prognosis of healing progress and monitoring response to antibiotic treatment in BUD patients.

For most mycobacterial infections, including tuberculosis, acquired cellular immunity is important for protection, but cellular immune responses are not well defined in BUD. It is known that up to one-third of lesions can heal spontaneously [16–18] and formation of granulomas has been reportedly associated with an induction of a proinflammatory and a down-modulation of inhibitory immune responses likely affecting the number of bacilli in the lesions [19, 20]. Therefore, it has been suggested that cellular immunity plays an important role in the immune response against *M*. *ulcerans*.

So far only few studies exist characterising immune responses in the context of *M*. *ulcerans* infection [21–25]. For instance, a broad analysis of systemic serum chemokines and cytokines revealed suppression of proteins including macrophage inflammatory protein (MIP)-1β, monocyte chemoattractant protein (MCP)-1 and IL-8 indicating immune modulation during *M*. *ulcerans* infection [26]. Immune responses to *M*. *tuberculosis* infections strongly depend on T helper type 1 cytokines IFNγ and tumor necrosis factor alpha (TNFα). Counteracting, regulatory immune responses based on the inhibitory cytokines Interleukin (IL-)10 and TGF-β are of major importance in several infectious diseases including tuberculosis [27]. In lesions of BUD patients, the key cytokines IFNγ and TNFα and the inhibitory cytokines IL-10 and TGF-β are produced, but the relative expression of these cytokines varied with the stage of the disease [20]. Modulated concentrations of TNFα were reported in serum of BUD patients if compared to controls [28]. Higher levels IFNγ and IL-10 were detected in BUD patients compared to household contacts or non-endemic controls following stimulation with *M*. *ulcerans* sonicate, in a study focusing on IFNγ and IL-10 from supernatants of whole blood [29]. However, there are no studies investigating proportions of cytokine producing CD4+ T cells in BUD patients, which allow dissecting regulation of specific cytokine-producing CD4 T cell subsets in the context of BUD, including multiple cytokine producing CD4+ T cells or T cells producing a single cytokine (based on what has been measured).

The analysis of specific cytokine producing CD4+ T cells in active tuberculosis and latently infected tuberculosis patients revealed differences in expression of CD40L- T cells [30]. Expression of CD40L on CD4+ T cells has been associated with a T_H_1 signature in infections with *M*. *tuberculosis* [31, 32]. Elsewhere, differences in IFNγ+TNFα+IL-2+, TNFα-single positive CD4+ T cells or a general increase in multi-cytokine producing T cells expression were observed between tuberculosis patients and contacts [33, 34]. Higher proportions CD40L+IL-2- CD4+ T cells were associated with cystic fibrosis patients infected with *Mycobacterium abscessus* [35], highlighting the potential of characterizing the precise profile of cytokine produced by CD4+ T cells in mycobacterial infections.

Therefore the aim of this study was to analyse the profile and frequencies of cytokine producing CD4+ T cells after stimulation with *M*. *ulcerans* antigen in BUD patients and healthy contacts and to analyse specific cytokine-secreting CD4+ T cell subsets in the context of disease severity and progression.

## Materials and methods

### Ethical approval

Ethical approval of the study was obtained from the ‘Committee on Human Research, Publication and Ethics (CHRPE) at the School of Medical Sciences, Kwame Nkrumah University of Science and Technology, Kumasi, Ghana (Ref.: CHRPE/AP/275/14 and CHRPE/AP/301/15). In addition, ethical approval was granted by the Ethical committee of the medical faculty of the Heinrich-Heine-University Dusseldorf (Ref.: 3903). The aims and procedures were explained to participants and/or their parents/guardians prior recruitment into the study. Only compliant patients were recruited and they were free to withdraw at any point during the study. Written consent was obtained from the parents or guardians. In some cases (illiterate guardians/parents) consent was confirmed by thumbprint, a procedure approved by the review board.

### Study population, recruitment and selection criteria

Between September 2014 and February 2016, patients with BUD were recruited at the Agogo Presbyterian Hospital in the Asante Akim North District, where there is high incidence of BUD in the middle forest belt of Ashanti region of Ghana [36].

A patient was recruited when the presenting lesion was consistent with the WHO clinical disease definition for BUD and diagnosis later confirmed by *M*. *ulcerans* IS2404 PCR. This study is part of a larger study investigating immune modulation in BUD patients with and without concomitant co-infections in children and adolescence. Up to 50% of all BUD cases in Africa are diagnosed in children below the age of 17 years [1, 37] with age being a significant factor for the clinical presentation of the disease [38]. The present study is specifically focusing on children and adolescent. Participants had to be ≤ 17 years of age, to be recruited into the study. Fifty-one of 101 (50.5%) patients were excluded for falling outside the age limit. Patients were also excluded if they *i)* had a history of BUD, tuberculosis or leprosy, *ii)* had a history of liver or kidney diseases, *iii)* were HIV positive (none excluded) or tested positive for any helminth infection, *iv)* had a recent or current antibiotic use. A flow chart indicating selection of the study group is shown in S1 Fig.

A control group of age- and gender matched healthy contacts was recruited from siblings, relatives or household contacts of participants. All participants were asked about their presenting active BUD disease, family history of BUD exposure, their previous medical history, household demographics and previous treatments.

### Diagnosis and treatment

Clinical BUD cases were confirmed by PCR for the *IS2404* repeat sequence specific for *M*. *ulcerans* [39]. Lesions were classified by their form: nodule, oedema, plaque or ulcer. Lesion size was determined by measuring the widest diameter of a lesion and surface area lesion in cm^2^ (to take into account differences in the shape of lesions). Lesions were categorized following WHO guidelines: Category I: lesion <5cm in the widest diameter, Category II: lesion <15 cm, category III: lesion >15 cm, multiple lesions, osteomyelitis. Stool samples were taken for diagnosis of soil-transmitted helminths (STH) and infection with *S*. *mansoni* using the Formol-Ether concentration method. Microfilariae were detected using Sedgewick chamber using 100 μl of whole blood [40]. Depending on the initial result, up to 1000 μl of blood was filtered on a nucleopore filter membrane (3 μm) (Whatmann). Filters were stained with Giemsa and analysed by microscopy [40]. Malaria (*Plasmodium falciparum*) status was determined using the rapid test CareStart Malaria HRP2 pf (Access Bio, Inc.). Haematological parameters were assessed using the Sysmex XS-800i system (Sysmex).

Patients with BUD received a standard treatment regime with 15 mg/kg streptomycin and 10 mg/kg rifampicin daily for 8 weeks, as recommended by the WHO [7]. Patients presented every two weeks during antibiotic treatment and monthly subsequently for monitoring of healing progress until complete healing.

### Immunological assays and flow cytometry

Up to 10 mL of venous blood was collected into heparinised blood collection tubes (BD Biosciences) between 9am and 12noon, prior initiation of antibiotic treatment. Blood samples were transported to the laboratory based in Kumasi and immediately processed (in less than 6 hours after blood has been taken [41, 42]). Surface stains to identify T, B, NK and CD16+ myeloid cells was performed directly in 100 μl of whole blood, diluted with 100 μl RPMI 1640 supplemented with 100 U/mL penicillin and 100 μg/mL streptomycin. Following incubation with the fluorochrome-conjugated antibodies for 30 min, red blood cells were lysed using a red blood cell lysis buffer (Roche) and remaining leucocytes were fixed for 15 min using Cytofix Solution (Biolegend).

For detection of intracellular cytokines, 100 μl of whole blood was stimulated in 96 well round bottom plates with *M*. *ulcerans* antigen at a final concentration of 5 μg/mL or with Staphylococcal enterotoxin b (SEB; Sigma) at a final concentration of 15 μg/mL. *M*. *ulcerans* antigen sonicate was prepared from a *M*. *ulcerans* 1 isolate of African origin. The origin and preparation of the *M*. *ulcerans* antigen are described in detail elsewhere [29].

Stimulated blood was incubated for a total of 17.5 hr at 37°C, 5% CO_2_ and Brefeldin A (Sigma) was added after an initial incubation of 2.5 hr. Whole blood cultured in medium without any stimuli was used as unstimulated negative control. Following incubation, red blood cells were lysed and the remaining cells fixed and permeabilized using a permeabilization Wash Buffer (Biolegend). Fluorochrome-conjugated antibodies (CD4, TNFα, IFNγ, CD40L) were added and incubated for 30 min followed by two additional wash steps.

Stained blood was acquired on a BD Accuri C6 Flow Cytometer (BD Biosciences) and analysed using FlowJo v10 (TreeStar). For further analysis, *M*. *ulcerans* or SEB-specific responses were determined by subtracting unstimulated control values from SEB or *M*. *ulcerans* stimulated cells. If subtracted values were ≤ 0, values were set to 0.001 for illustration purposes.

Following anti-human antibodies were used for flow cytometric analysis: APC-conjugated CD16 (clone 3G8), PerCP-Cy5.5.-conjugated CD3 (HIT3a), FITC-conjugated CD56 (HCD56), Alexa488-conjugated CD4 (RPTA-4), APC-conjugated TNFα (MAB11), PerCP-Cy5.5.-conjugated CD154/CD40L (24–31; all Biolegend), PE-conjugated IFNγ (2572311, BD Bioscience) and PE-conjugated CD20 (2H7, eBioscience). All staining panels were evaluated using fluorescence minus one and unstained controls.

### Statistical analyses

Since analysed data were not normally distributed (Shapiro-Wilk) and did not meet assumptions for parametric tests, non-parametric tests were applied. For comparison of two groups (e.g. BUD patients *vs*. contacts) Mann-Whitney *U* test was used. Correlations were tested using Spearman’s rho analysis. Statistical analyses were performed using SPSS v23 (IBM Corp.) and were taken as significant if ≤ 0.05. Graphical illustration was done using Graphpad v7.0 (GraphPad Software, Inc.).

Lesion types were separated into groups based on their lesion form: non-ulcerative forms (nodule/oedema/plaque) and ulcerative forms. Patients were also separated into two groups based on the time to healing following the start of antibiotic treatment, using a cut-off of 111 days (based on the median healing time = 111.0 days, range 14–337 days) or alternatively based on the time healing was first recognized (cut-off based on the median of 56 days, range 14–231 days).

## Results

### Description of study groups

Blood samples were obtained from 36 BUD patients with a median age of 8.5 years (range 1.5–17 years) and from healthy contacts (median age 7.0 years; range 3.0–15 years). Detailed characterisation of the study groups are provided in Table 1. Haematological parameters were compared between BUD patients and contacts. There was a moderate, but significant increase in the frequency of basophils (Table 1). There was no difference between major lymphocyte subsets including T cells, B cells and NK cells (S2A–S2C Fig), but proportions of CD16+ myeloid cells were increased in BUD patients (S2D Fig). Additional haematological parameters did not differ between the two groups (Table 1). Clinical characteristics of the BUD group are shown in Table 2. Lesion size varied between individual BUD patients (2.3–79.4 cm^2^). Based on the widest diameter all lesions but one were classified as category I or II lesions (for details see methods section). One lesion with 15.7 cm in the widest diameter was classified as category III lesion. The majority of patients presented with either nodules (50.0%) or with ulceration (30.6%) (Table 2). None of the patients included into the study presented with multiple lesions or osteomyelitis.

**Table 1:** Description of the study population.

	BUD patients	BUD contacts	p
**N**	36	22	
**Mean age (range)**	8.1 (1.5–17)	7.8 (3–15)	0.981
**Median age**	8.5	7.5	
**Female / male (% males)**	14/22 (61.1)	12/10 (45.5)	0.249
**Malaria status +/- (% positive)**	17/18* (48.6)	11/11 (50.0)	0.917
**BCG scar +/- (% positive)**	25/10* (71.4)	20/2 (90.9)	0.103
***mean +/- SEM***	***Haematological parameters***		
N**	31	21	
**WBC** (10^9/L)	6.95 +/- 0.48	5.74 +/- 0.74	**0.050**
**RBC** (10^12/L)	4.13 +/- 0.11	4.31 +/- 0.22	0.880
**HB** (g/dL)	10.79 +/- 0.24	11.56 +/- 0.56	0.385
**HCT** (%)	32.66 +/- 0.88	32.77 +/- 1.84	0.636
**MCV** (fL)	78.94 +/- 1.26	75.17 +/- 1.40	0.107
**MCH** (pg)	25.11 +/- 0.43	26.87 +/- 0.93	0.124
**PLT** (10^9/L)	237.56 +/- 26.49	238.00 +/- 22.43	0.654
**Lymphocytes** (%)	44.44 +/- 2.38	46.40 +/- 3.65	0.449
**Monocytes** (%)	10.66 +/- 0.74	10.79 +/- 1.13	0.538
**Neutrophils** (%)	33.69 +/- 2.42	30.79 +/- 2.74	0.526
**Eosinophils** (%)	6.98 +/- 1.15	4.95 +/- 1.28	0.106
**Basophils** (%)	3.15 +/- 1.28	2.31 +/- 0.70	**0.049**

*one patient with unknown status;

**N = 5 and N = 1 data are not available

**Table 2:** Clinical characteristics of the BUD group.

Clinical parameter	Measurement	
Duration of disease*	N	35
	Mean (range) in weeks	3.4 (1–8)
**Lesion size**	N	31
	Mean surface (range) in cm^2^	22.1 (2.3–79.4)
	Mean volume (range) in cm^3^	0.59 (0–12)
**Lesion Category**	Type I (< 5 cm**) N (%)	16 (51.6)
	Type II (< 15 cm**) N (%)	14 (45.2)
	Type III (> 15 cm**) N (%)	1 (3.2)
**Lesion type**	N	36
	Nodule N (%)	18 (50.0)
	Oedema N (%)	2 (5.6)
	Plaque N (%)	5 (13.9)
	Ulcer N (%)	11 (30.6)
**Time to healing** (following start of antibiotic treatment)	N	34
	Mean/Median (range) in days	115.9/111.0 (14–337)
	fast ≤ 111 days N (%)	17 (50%)
	slow > 111 days N (%)	17 (50%)
Rate of healing***	N	26
	Median (range) in mm/week	0.44 (-0.94–1.8)

* time between recognizing first symptoms and reporting at the hospital

** widest diameter

*** in the first 4 weeks

### BUD patients show increased T_H_1 cytokine producing CD4+ T cells

**Fig 1:**
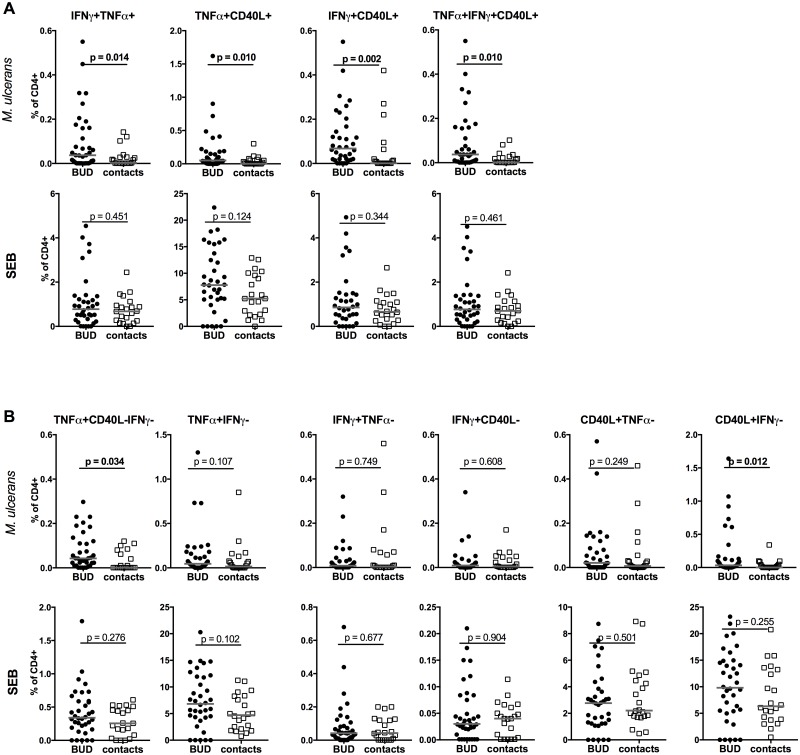
T_H_1 cytokine producing CD4+ T cells in Buruli ulcer disease patients and healthy contacts. Whole blood was cultured for 17.5 hrs with either *M*. *ulcerans* antigen or Staphylococcal enterotoxin b. Cells were analysed by flow cytometry for TNFα, IFNγ and CD40L producing CD4+ T cells. Proportions of double and triple cytokine producing T cells (IFNγ+TNFα+, TNFα+CD40L+, IFNγ+CD40L+ and IFNγ+TNFα+CD40L+) are indicated in (**A**). Combinations of cytokine producing CD4+ T cells are shown in (**B**). Medians are indicated in grey and groups are compared by non-parametric Mann-Whitney *U* test and p values indicated.

To determine the specific cytokine producing profile of *M*. *ulcerans* specific CD4+ T cells, whole blood samples were stimulated overnight with *M*. *ulcerans* antigen and analysed for TNFα, IFNγ and CD40L producing T cells by flow cytometry. An example of the according gating and cytokine production is shown in S3 Fig. BUD patients had significant higher proportions of CD4+ T cells producing two (IFNγ+TNFα+, TNFα+CD40L+, IFNγ+CD40L+) or all three (IFNγ+TNFα+CD40L+) cytokines when compared to contacts (Fig 1A, upper panels). Only a minor fraction of the BUD contacts showed detectable levels of these cytokine-producing cells in response to *M*. *ulcerans* antigen. In addition, frequencies of two subsets characterised as TNFα+CD40L- and CD40L+IFNγ-, were increased in BUD patients (Fig 1B, upper panels). Further characterisation of these subsets revealed, that TNFα+CD40L- CD4+ T cells were almost exclusively IFNγ- (median of IFNγ- = 100%) and were therefore denoted as TNFα+CD40L-IFNγ-. In contrast, CD40L+IFNγ- contained both TNFα+ and TNFα- cells (median of TNFα+ = 42.9%). Neither CD40L+IFNγ-TNFα- nor IFNγ+CD40L-TNFα- single positive CD4+ T cells differed between BUD patients and contacts (p = 0.134 and p = 0.881 respectively).

No significant differences between both groups were observed following stimulation with Staphylococcal enterotoxin b used as polyclonal control stimulation (Fig 1A and 1B, lower panels). In summary, CD4+ T cells of BUD patients showed specific cytokine profile characterised by induction of multiple cytokine producing cells as well as an increase in the frequency of TNFα+CD40L-IFNγ- CD4+ T cells.

### TNFα+CD40L-IFNγ- CD4+ T cells are correlated with lesion size

**Fig 2:**
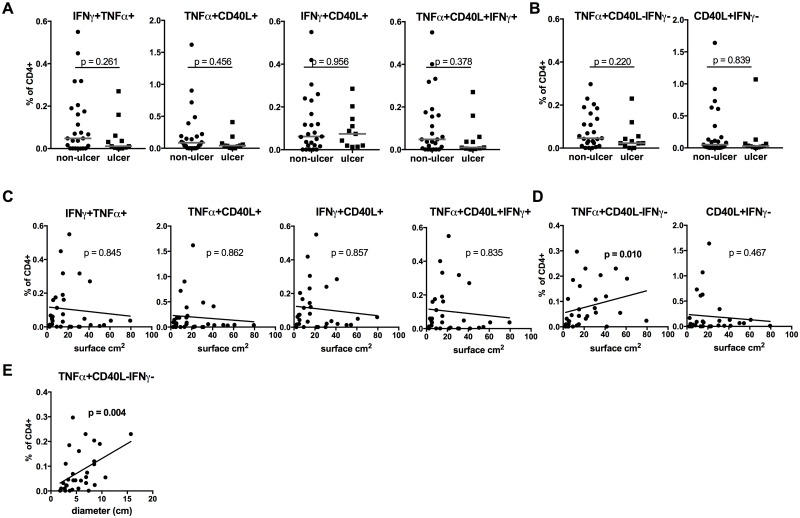
TNFα+CD40L-IFNγ- CD4+ T cells are correlated with size of lesions in Buruli ulcer disease. Cytokine producing CD4+ T cells were determined as for Fig 1 and analysed in regards to type of lesion (**A**, **B**) or surface area of lesions **(C**, **D**) or widest diameter of the lesions (**E**). Multiple cytokine producing are shown in (**A**, **C**); TNFα+CD40L-IFNγ- and CD40L+IFNγ- are indicated in (**B**, **D**). P—values of non-parametric Mann-Whitney *U* analyses (A, B) and Spearman correlations (C–E) are presented.

BUD patients presented at different stages of disease characterized by different types of lesions and varying lesion sizes (Table 2). Cytokine producing CD4+ T cells were analysed in the context of these clinical presentations to determine their potential as biomarkers. Data were split into two groups comprising patients with non-ulcerative forms (nodules, oedema, plaque) or ulcer. Neither multiple cytokine producing CD4+ T cells producing TNFα+IFNγ+, TNFα+CD40L+, TNFα+IFNγ+CD40L+ differed between the two groups (Fig 2A) nor TNFα+CD40L-IFNγ- CD4+ T cells showed any differences (Fig 2B). However, the size of lesions did not differ significantly between these two groups (p = 0.508). Therefore the lesion size was correlated with cytokine producing CD4+ T cells. There was no correlation between multiple cytokine producing T cells (Fig 2C). However, TNFα+CD40L-IFNγ- CD4+ T cells were positively correlated with surface area of the lesion (rho = 0.456; p = 0.010) (Fig 2D), which was reflected by a significant correlation with the widest diameter of the lesion (rho = 0.529; p = 0.004) (Fig 2E). None of the additional CD4+ T cell subsets presented in Fig 1B (TNFα+IFNγ-, IFNγ+TNFα-, IFNγ+CD40L-, CD40L+TNFα-) was correlated with lesion size (S4 Fig).

**Fig 3:**
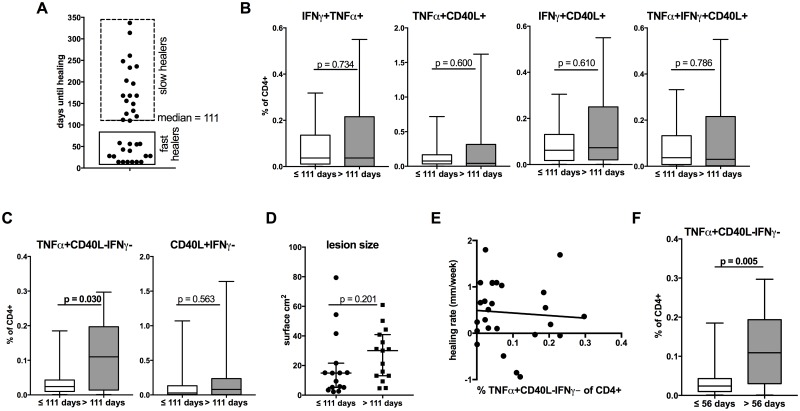
TNFα+CD40L-IFNγ- CD4+ T cells differ between fast and slow healers in Buruli ulcer disease. (**A**) BUD patients were divided into fast healers (≤ 111 days) and slow healers (> 111 days) based on the time required for complete healing and proportions of cytokine producing CD4+ T cells (**B**, **C**) or lesion sizes (**D**) are compared by non-parametric Mann Whitney *U* test. The healing rate within the first four weeks (in change if mm/week) was correlated with TNFα+CD40L-IFNγ- CD4+ T cells (**E**) by Spearman correlation or compared based on time until healing process starts after initiating antibiotic treatment (**F**).

Patients were treated with a combination of rifampicin and streptomycin for eight weeks and time until complete healing was monitored. Applying the healing time, patients could be divided into ‘fast’ and ‘slow’ healers (Fig 3A). A cut-off of 111.0 days (median healing time) was applied to classify and distinguish these two groups. Notably, we detected significantly increased proportions of TNFα+CD40L-IFNγ- CD4+ T cells in ‘slow’ compared to ‘fast’ healers, whereas none of the other cytokine producing subsets were significantly different between the two groups (Fig 3B and 3C). Of note, ‘fast’ and ‘slow’ healers did not differ significantly by age (p = 0.193) or gender (p = 0.079) or original lesion size (Fig 3D). There was no significant correlation between healing rate within the first four weeks (expressed as mm/week) and TNFα+CD40L-IFNγ- CD4+ T cells (Fig 3E).

Some lesions do not immediately start to heal following initiation of antibiotic treatment, a phenomenon that has been described in BUD patients. For BUD patients included in this study healing started at a median duration of 56 days and this value was used to distinguish two groups. BUD patients with lesion starting to heal in less than 56 days had significant lower proportions of TNFα+CD40L-IFNγ- CD4+ T cells compared to patients with lesions starting after more than 56 days (Fig 3F).

Of note, is the fact that TNFα+CD40L-IFNγ- CD4+ T cells did not differ between BCG scar positives and negatives (p = 0.815).

## Discussion

In the present study, we show that the TNFα+CD40L-IFNγ- CD4+ T cell subset, induced by *M*. *ulcerans* antigen, provides a promising approach for establishing an immune-based assay for monitoring BUD.

We evaluated the expression of T helper type 1 associated cytokines in combination with CD40L following stimulation with *M*. *ulcerans* sonicate. In particular multiple cytokine expressing CD4+ T cells (e.g. IFNγ+TNFα+, TNFα+CD40L+, IFNγ+CD40L+) were induced in BUD patients compared to healthy contacts. In addition, proportion of TNFα+CD40L-IFNγ- were also higher in BUD patients making it a unique single cytokine producing subset. Both multiple cytokine producing CD4+ T cells as well as TNFα single positive T cells have been identified to discriminate between latent and active tuberculosis [33]. TNFα-single positive T cells were also the strongest predictor of an active tuberculosis in a larger study [43]. In BUD this unique TNFα-single positive T cell subset may reflect a strong inflammatory rather than a protective response. However, the functional role of TNFα+CD40L-IFNγ- CD4+ T cells in BUD needs to be investigated in more detail.

Diagnostic tests based on immunological responses are routinely used for detecting an infection with *M*. *tuberculosis*. IGRA’s are based on the antigen-induced production of IFNγ, but comparable assays are not available for BUD. Our findings may provide an approach to develop such an immune-based assay.

In BUD patients, suppression of immune responses can be recognized locally within lesions [20, 21, 44]. Whether a generalised systemic immune suppression can be detected is not yet clarified with contradictory findings in different studies [21, 23, 29, 45]. In our study, none of the analysed cytokine-producing CD4+ T cell subsets were lower in BUD patients compared with contacts suggesting that patients are able to mount T_H_1 responses confirming earlier results [29]. If immune suppression can be detected systemically as found by others [21, 46], may depend on the stage of disease, time of sampling, stimulation and method of analysis.

As there is currently no vaccine for BUD and the mode of transmission remains unclear disease management and optimization of treatment will remain highly important for the foreseeable future. Therefore we evaluated cytokine producing CD4+ T cells in the context of several clinical characteristics of BUD. First we compared BUD patients with different types of lesions (ulcer *versus* non-ulcerative forms). None of the analysed subsets including TNFα+CD40L-IFNγ- CD4+ T cells differed between the two groups.

Since the type of lesions was not necessarily associated with the size of lesion, we also correlated our identified cytokine producing CD4+ T cells subsets with surface area and with the widest diameter of lesions. Interestingly the only parameter showing a correlation was the TNFα+CD40L-IFNγ-, further supporting the role of this subset as biomarker.

An effective treatment based on rifampicin and streptomycin has been established and is recommended by the World Health Organization [1, 7]. The efficacy of the treatment regime is more than 90% [47, 48]. Nevertheless the healing process varies widely. Healing may start directly upon initiation of antibiotic treatment or it may take several weeks until healing becomes obvious and time until complete healing is obtained varies considerably. In addition, lesions may increase in size during or following initiation of antibiotic treatment, a phenomenon, which is mainly attributed to recovery of immune responses and is referred to as ‘paradoxical reaction’ [49–51]. Such paradoxical reactions are reported in more than 20% of cases [49, 52]. Given this, immunological markers, which help to predict healing and potentially ‘paradoxical reactions’ could be beneficial in terms of patient care and optimisation of antibiotic treatment. Since paradoxical reactions are thought to be based on changes in immune response [53], identifying biomarkers is a promising approach.

In the present study complete healing was observed between 14 and 337 days (median 111.0), which is in the range of observed in other studies [47, 48]. Comparing ‘slow’ *versus* ‘fast’ healers revealed indeed that only TNFα+CD40L-IFNγ- expression differed between the two groups. The difference in time to healing could theoretically be attributed to the fact that larger lesions need longer time for complete healing. However, in our study, the size of lesions did not differ significantly between ‘slow’ and ‘fast’ healers. Therefore the fact that TNFα+CD40L-IFNγ- differed between the two groups could not solely be attributed to an association with the original lesion size. In addition the healing rate within the first four weeks after start of antibiotic treatment did not correlate with TNFα+CD40L-IFNγ-. The time until first signs of healing were also recorded and varied between 14 and 231 days (median 56 days), consequently we analysed two groups based on this median. Indeed, the frequency of TNFα+CD40L-IFNγ- CD4+ T cells was higher in patients, which showed a delayed start of healing. Evaluating the healing progress using biomarkers may also be of importance in the evaluation of novel treatment regimens, such as full oral therapies, which are currently tested [54]. Of note, we did not have sufficient numbers of patients with a paradoxical reaction (N = 3) to evaluate differences in cytokine producing CD4+ T cells as prognostic marker for this. Therefore it needs to be further analysed if TNFα+CD40L-IFNγ- T cells are useful in this context in a larger study with more patients included.

The current study is focusing on children and adolescent. Age is an important factor affecting the clinical presentation of BUD [38] and in Africa more than 50% of all BUD cases are diagnosed in children. Therefore this age group would benefit most of improved biomarkers. However, in addition, TNFα+CD40L-IFNγ- producing CD4+ T cells as biomarker has to be tested in older BUD patients, which are more likely to develop severe forms of BUD [55].

In summary, we present here the first study analysing cytokine producing CD4+ T cells stimulated with *M*. *ulcerans* antigen focusing at T helper type 1 CD4+ T cells. Proportions of multiple cytokine as well as TNFα+CD40L-IFNγ- CD4+ T cells differed between BUD patients and healthy contacts and the later one was associated with lesion size and differed between ‘slow’ and ‘fast’ healers. Hence TNFα+CD40L-IFNγ- CD4+ T cells are a potential biomarker, which may allow the development of tests comparable to IGRAs.

## Supporting information

S1 Fig Flow diagram. The flow diagram indicates the recruitment procedure and exclusion criteria of the presented study.
(PDF)

S2 Fig Leucocyte subsets in Buruli ulcer patients. Whole blood of BUD patients and contacts was analysed for T cells by CD3 expression (**A**), B cells by CD20 expression (**B**), NK cells by CD56 expression (**C**) and myeloid cells by CD16 expression (**D**) using flow cytometry. Proportions of lymphocytes (A-C) or total leucocytes (D) are indicated and compared using a non-parametric Mann-Whitney *U* test. Grey line indicates the median.
(PDF)

S3 Fig *M*. *ulcerans* specific cytokine production by CD4 T cells. Whole blood was cultured for 17.5 hrs in medium, with *M*. *ulcerans* crude antigen or SEB in the presence of Brefeldin A. Following red blood cell lysis, cells were gated on CD4+ T cells (**A**) and analysed for TNFα, IFNγ and CD40L. (**A)** and (**B**) show a sample of an 11 years old male BUD patient with low TNFα+CD40L- proportions, while (**C**) shows a BUD patient (11 year old male), with higher proportions of TNFα+CD40L- upon stimulation with *M*. *ulcerans* sonicate.
(PDF)

S4 Fig Additional cytokine producing CD4+ T cell subsets in correlation to the size of lesions. TNFα+IFNγ-, IFNγ+TNFα-, IFNγ+CD40L-, CD40L+TNFα- CD4+ T cell subsets were determined as described in Fig 1 and correlated to the surface area of lesions.
(PDF)
