# Supplementary figures and images for: Analysis of Mycobacterium ulcerans-specific T-cell cytokines for diagnosis of Buruli ulcer disease and as potential indicator for disease progression
Source: PLoS Negl Trop Dis. 2017 Feb 27;11(2):e0005415. doi: 10.1371/journal.pntd.0005415 (PMC5344519; doi:10.1371/journal.pntd.0005415)

S1 Fig

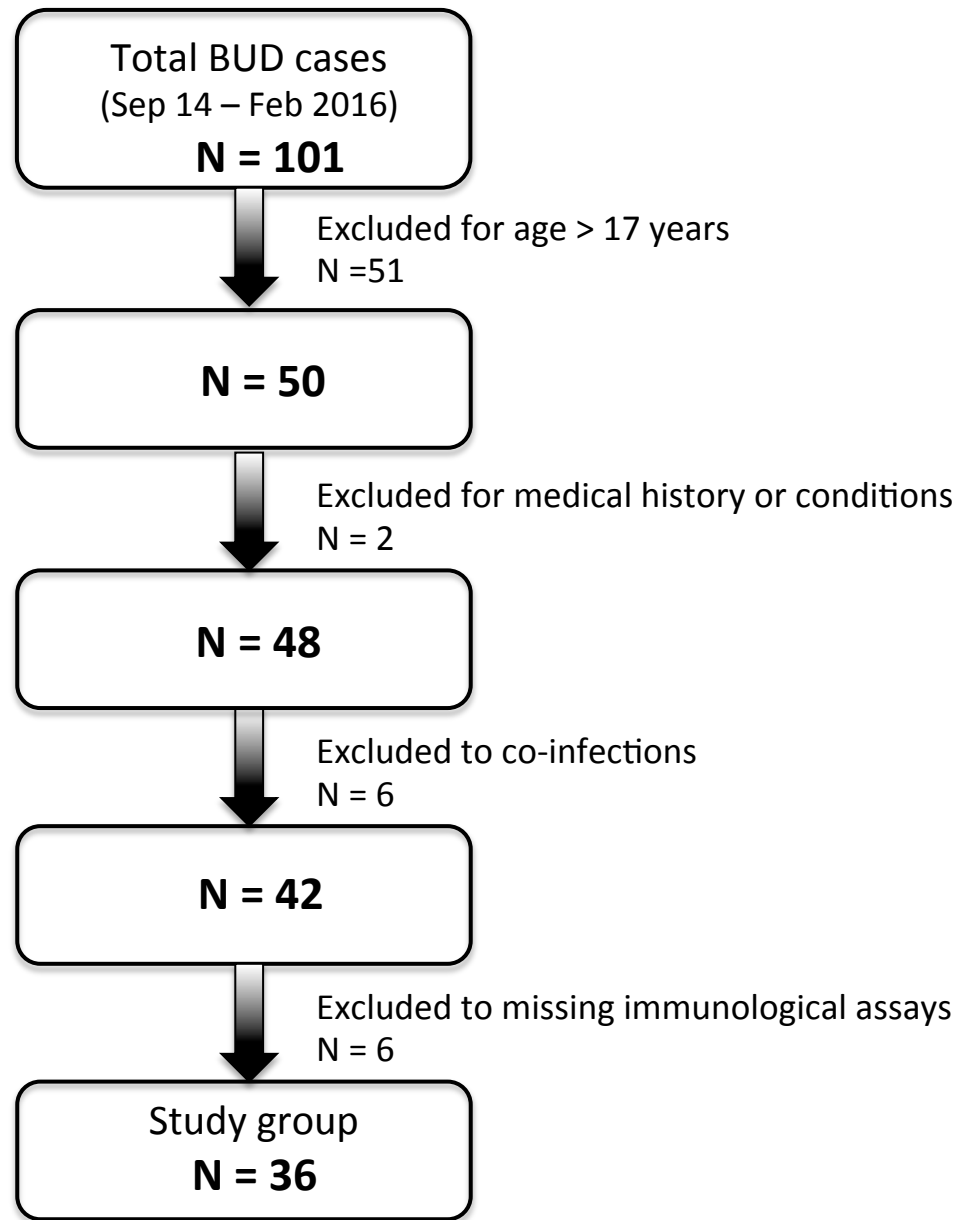

Supplement: S1 Fig — The flow diagram indicates the recruitment procedure and exclusion criteria of the presented study. (PDF) [file pntd.0005415.s001.pdf]

S2 Fig

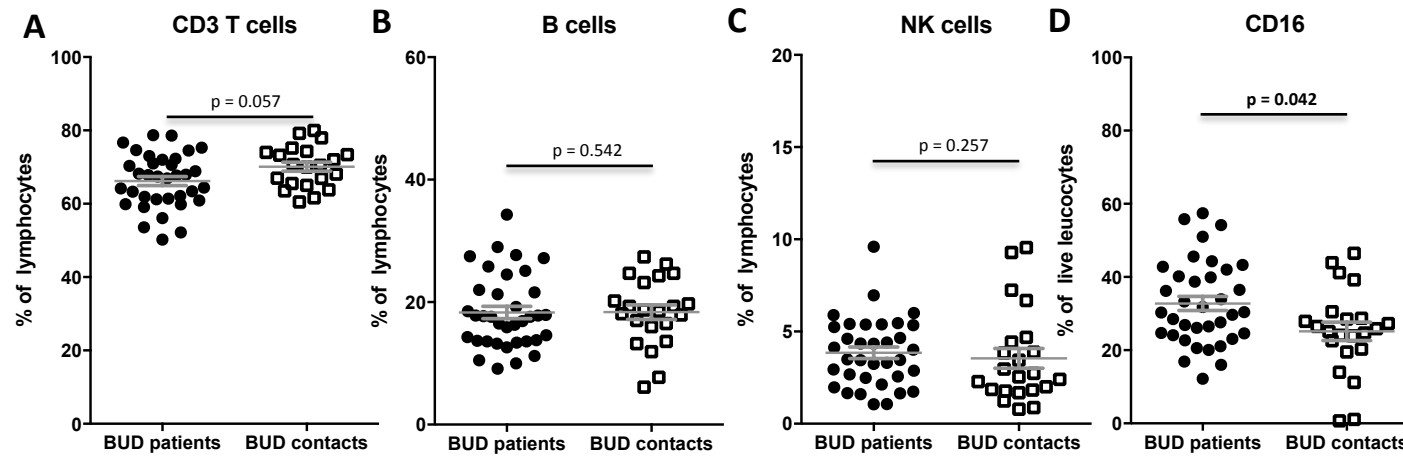

Supplement: S2 Fig — Whole blood of BUD patients and contacts was analysed for T cells by CD3 expression (A), B cells by CD20 expression (B), NK cells by CD56 expression (C) and myeloid cells by CD16 expression (D) using flow cytometry. Proportions of lymphocytes (A-C) or total leucocytes (D) are indicated and compared using a non-parametric Mann-Whitney U test. Grey line indicates the median. (PDF) [file pntd.0005415.s002.pdf]

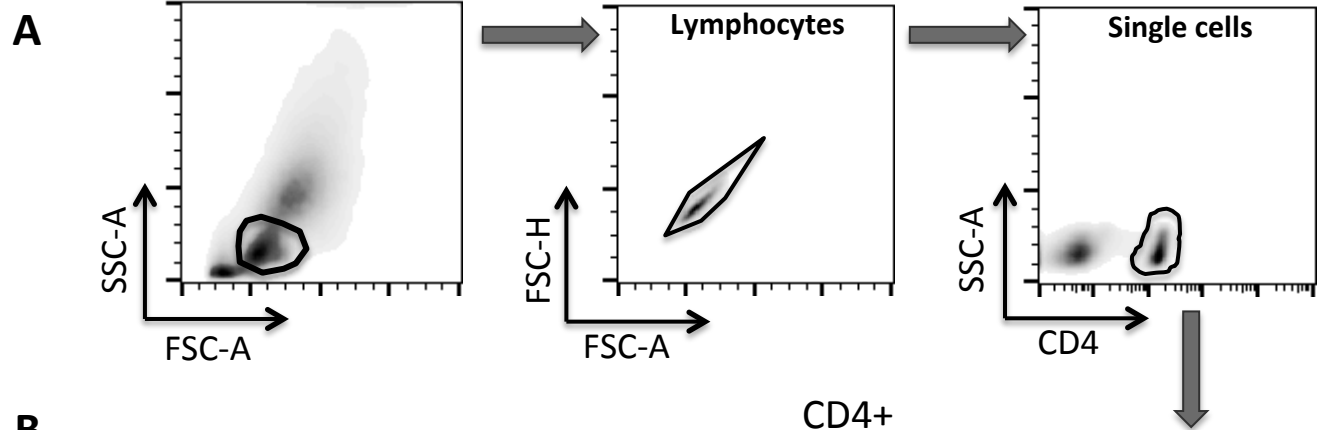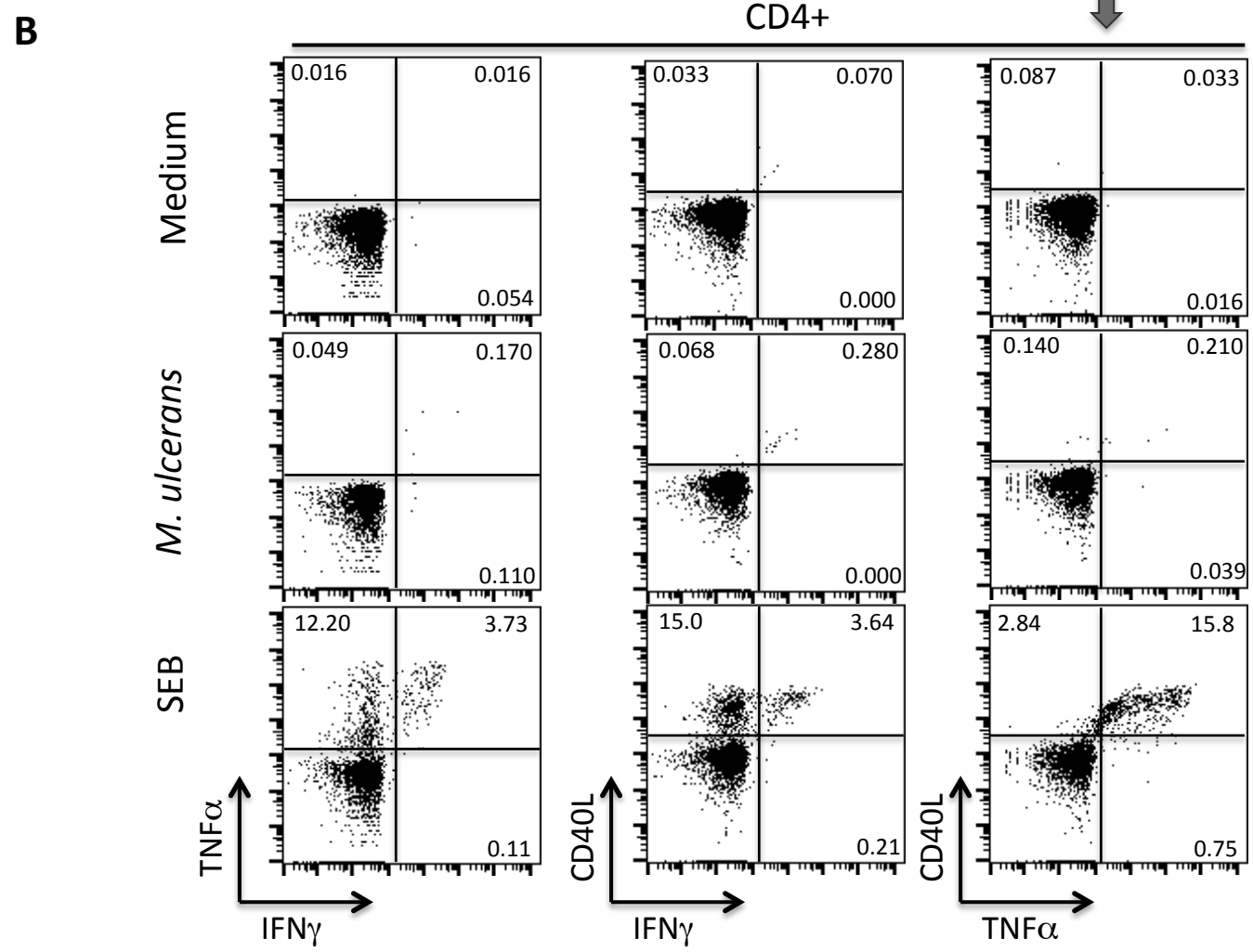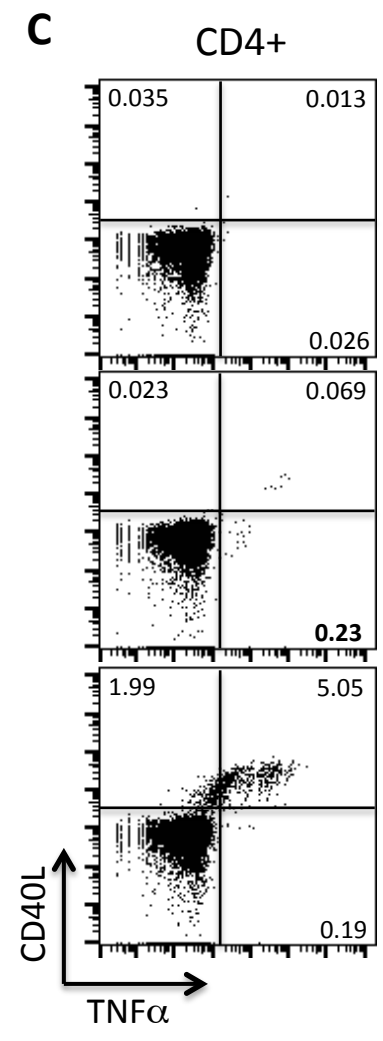

Supplement: S3 Fig — Whole blood was cultured for 17.5 hrs in medium, with M. ulcerans crude antigen or SEB in the presence of Brefeldin A. Following red blood cell lysis, cells were gated on CD4+ T cells (A) and analysed for TNFα, IFNγ and CD40L. (A) and (B) show a sample of an 11 years old male BUD patient with low TNFα+CD40L- proportions, while (C) shows a BUD patient (11 year old male), with higher proportions of TNFα+CD40L- upon stimulation with M. ulcerans sonicate. (PDF) [file pntd.0005415.s003.pdf]

S4 Fig

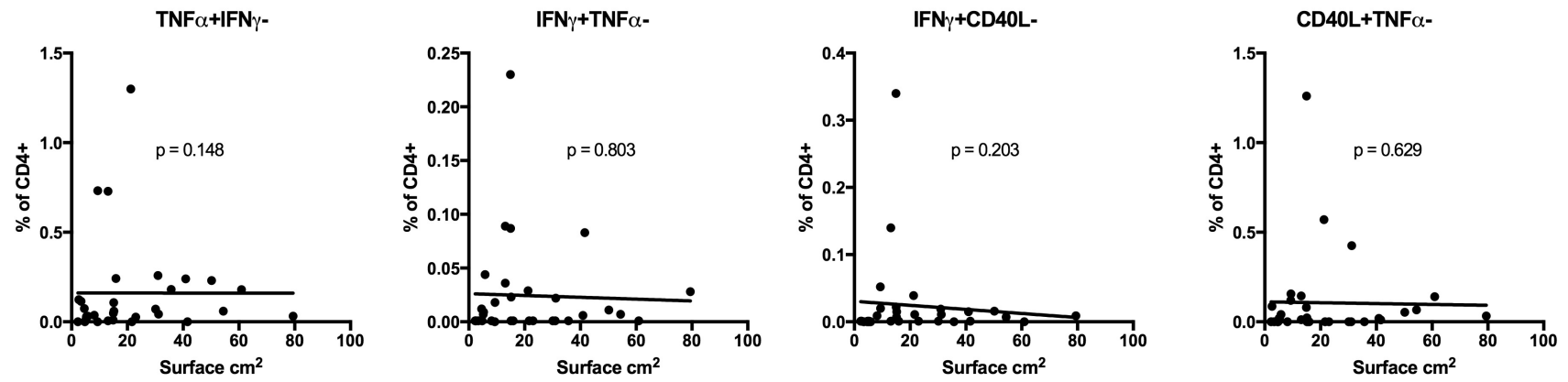

Supplement: S4 Fig — TNFα+IFNγ-, IFNγ+TNFα-, IFNγ+CD40L-, CD40L+TNFα- CD4+ T cell subsets were determined as described in Fig 1 and correlated to the surface area of lesions. (PDF) [file pntd.0005415.s004.pdf]
